# Correction: Phenylephrine induces relaxation of longitudinal strips from small arteries of goat legs

**DOI:** 10.1371/journal.pone.0257440

**Published:** 2021-09-10

**Authors:** Kawin Padmaja Marconi, Bhavithra Bharathi, Alen Major Venis, Renu Raj, Soosai Manickam Amirtham, Sathya Subramani

There are errors in Figs 2, 6, 9, and 11. The authors have provided corrected versions here.

**Fig 2:**
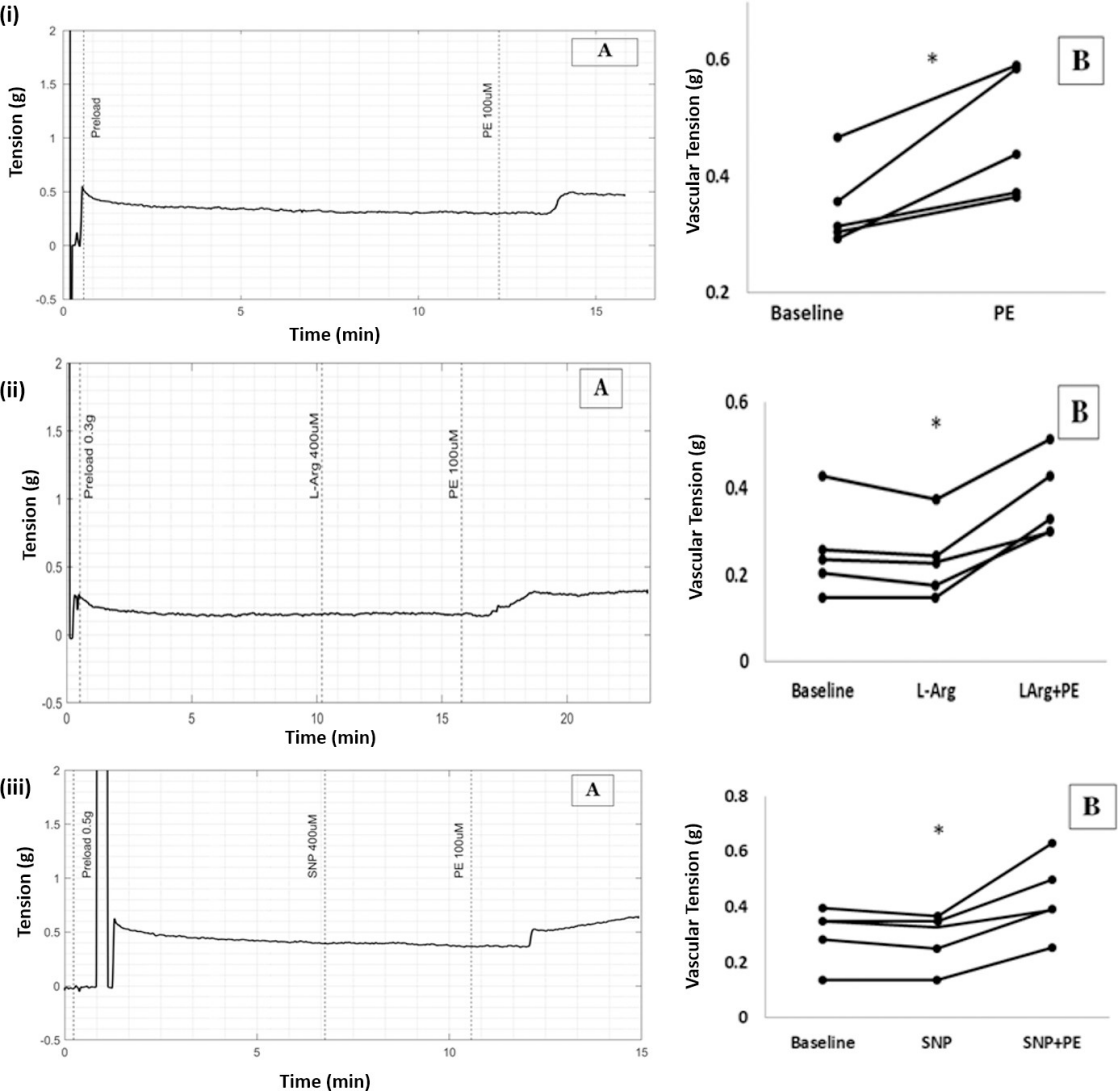
(A) Raw tracings showing changes in vascular tension in circular strips of aorta (B) Scatter plots of results from all five experiments demonstrating increase in vascular tension with PE. (C) Quantum of change with PE from baseline (* p< 0.05). (i) PE 100uM (ii) PE 100uM in high NO environment caused by L-Arginine (iii) PE 100uM in presence of SNP.

**Fig 6:**
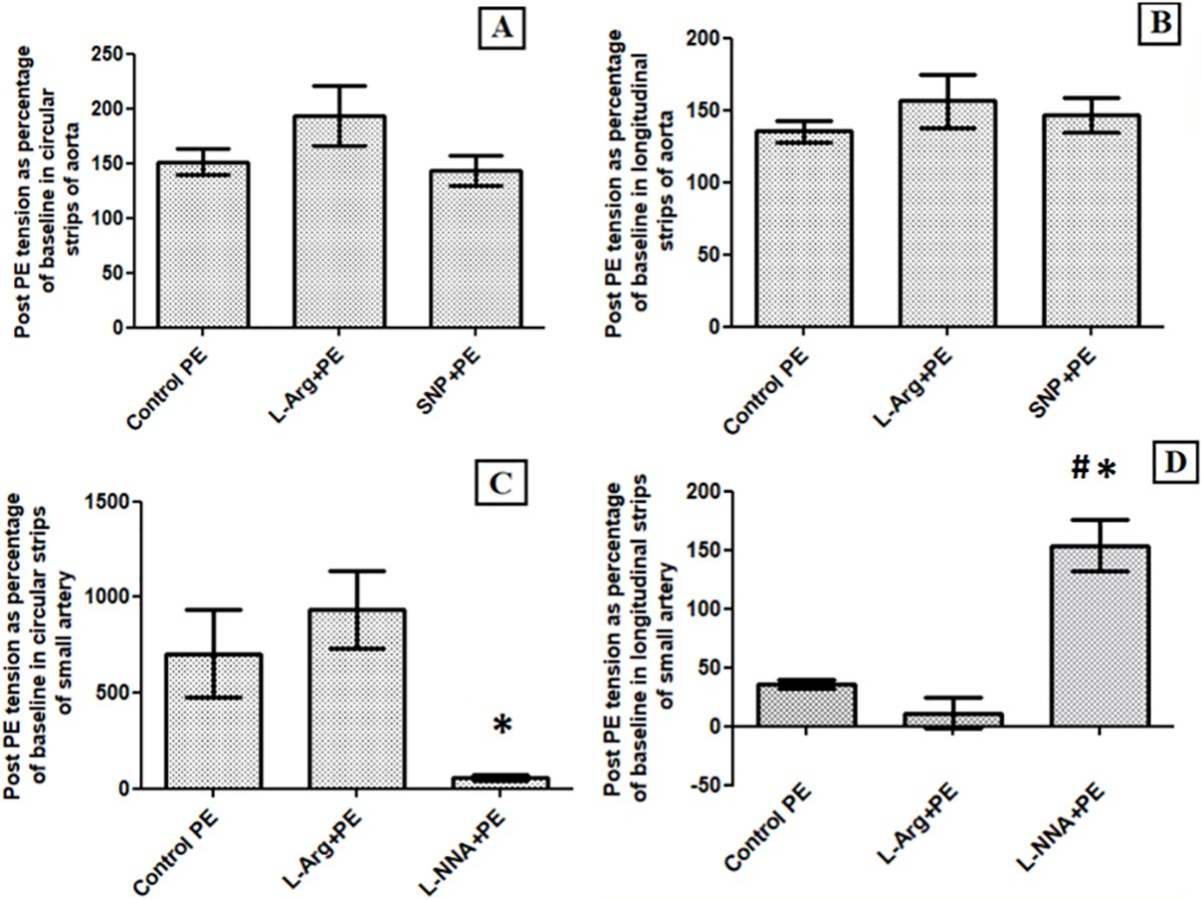
Bar diagrams representing tension after PE administration as a percentage of baseline tension (A) Circular Strip preparation of aorta, (B) Longitudinal strip preparation of aorta, (C) Transverse cylinder preparation of small artery and (D) Longitudinal strip preparation of small artery. There was no significant difference between the contractile responses to PE in the presence or absence of L-Arginine (P value = 0.273 with MWU) as well as SNP (P value = 0.754 with MWU) in circular and longitudinal strips of aorta. There was no significant difference in percentage change in tension due to PE in the presence or absence of L-Arginine in transverse cylinder preparation (P value = 0.273, MWU test) and longitudinal strip preparation of small artery (P value = 0.08, MWU test). In panel C, lack of contractile response of circular strips of small artery to PE (Post PE tension around 100% of baseline tension) in presence of L-NNA was statistically significant as compared to effect of PE alone (*). In panel D, the last bar represents contractile response to L-NNA itself, while there was no further increase in tension with addition of PE. Lack of relaxant response to PE was significant as compared to effect of PE alone.

**Fig 9:**
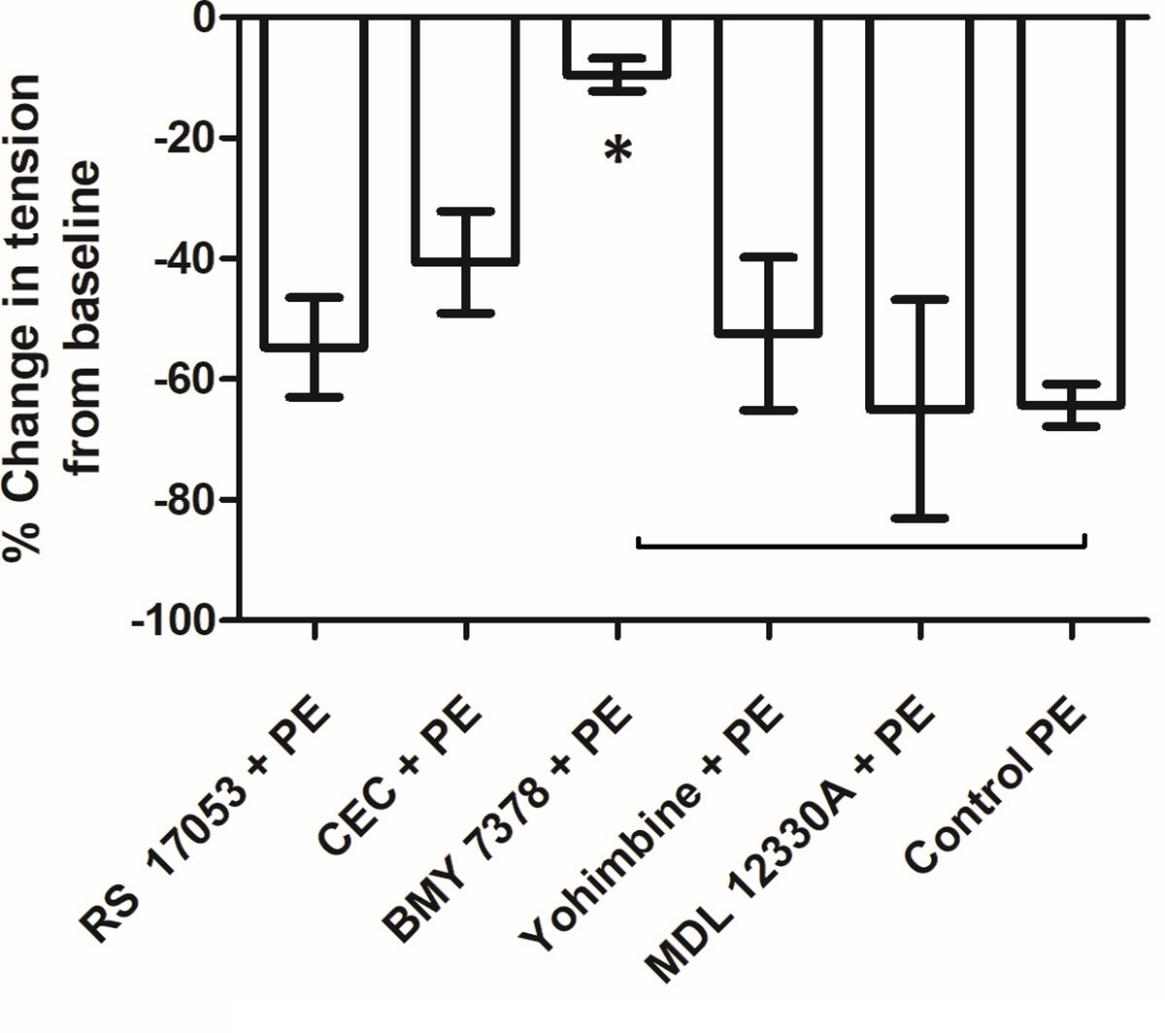
Bar diagram representing percent change in tension from baseline due to PE in the presence of blockers in longitudinal strip preparation of small artery. BMY 7378 an alpha 1D blocker, blocked the relaxant response to PE in a statistically significant manner (P value < 0.05 with MWU test).

**Fig 11:**
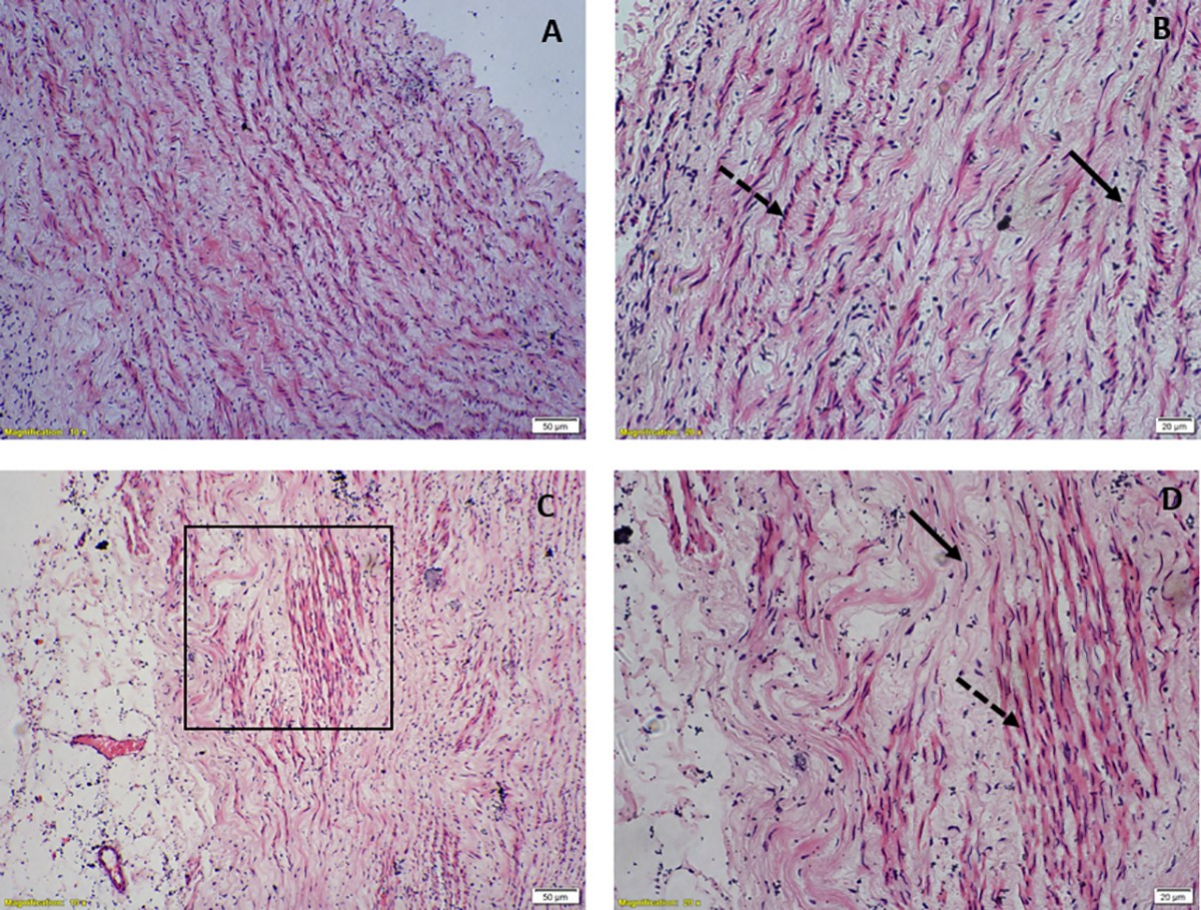
Hematoxylin & eosin stained light microscopic view of aorta, (A) & (B) Circular strip preparation showing elliptical nuclei of circular smooth muscle fibres (solid arrow) and round nuclei of longitudinal muscle fibres (dashed arrow) under 10X & 20X magnification respectively, (C) & (D) Longitudinal strip preparation showing elliptical nuclei of longitudinal smooth muscle fibres and round nuclei of circular smooth muscle fibres under 10X & 20X magnification respectively.
